# Professor W. D. Miller

**Published:** 1901-11

**Authors:** 


### PROFESSOR W. D. MILLER.

ONE of the interesting incidents connected with Professor Miller's recent visit to this country was a lecture given at the Department of Dentistry, University of Pennsylvania. The return to his Alma Mater was doubtless a pleasant experience to him, but to the large number of dentists and medical men from the city and surrounding country it was a revelation of what can be accomplished by intelligent and persistent labor.

The lecture in the main represented Dr. Miller's work in preparing sections of jaws and teeth, both human and of inferior animals. Those who have had experience in doing this work could appreciate the labor involved and the excellent results.

The novel feature of the work was not in the cutting, but in the very beautiful effects produced by staining, thus differentiating the various tissues and perfectly and beautifully exhibiting these through the electric lantern. This work is illustrated in the *Dental Cosmos* for October, but, while these illustrations are very beautiful, they do not compare in delicacy of coloring with those of the lantern exhibit.

One of the most interesting portions to the writer consisted of certain specimens that seemed to clear up several controversial points. Dr. Miller, very properly, did not dwell upon these, refraining from expressing any decided opinion. The first series of these specimens consisted of several slides, showing very beautifully the so-called, gelatinous plaques of Williams. The enamel was very perfectly shown covered with bacterial masses and without the slightest break in the tissue, except in one instance, where a previous abrasion had existed.

The question naturally arose in the minds of some of the audience, If gelatinous plaques, with the accompanying bacteria, give an acid reaction, as claimed by Williams and Black, should there not be some slight evidence that the enamel-rods were being gradually affected? So far as it went the exhibit was confirmatory of the opinion held by some that the acid character of the plaques had not been proved.

The second interesting exhibit was a slide remarkable in that it demonstrated the transparent zone of Tomes in two places in direct line with caries on one side of the section, and on another part a transparent zone where caries was absent, but where the tooth showed distinct marks of abrasion from wear in mastication. While this specimen could not settle the controversy maintained upon this subject since Tomes first announced the fact, it was quite confirmatory of the opinion held by Miller and others that the change in the dentine was the result of a slight irritation, in the first place by caries, and in the second by wear,—altogether a vital action in both instances.

It is to be hoped that Professor Miller will give the result of this portion of his work in future articles. It is a gratification to feel that his health is apparently very nearly restored to original conditions.

